# Acceptance and commitment therapy in rehabilitation for chronic pain and fatigue: a qualitative interview study with patients

**DOI:** 10.1080/02813432.2025.2608121

**Published:** 2026-01-06

**Authors:** May-Lill Johansen, Thor Eirik Eriksen, Ida Therese Solhaug

**Affiliations:** ^a^ Research Unit for General Practice, UiT The Arctic University of Norway, Tromsø, Norway; ^b^ Department of Community Medicine, UiT The Arctic University of Norway, Tromsø, Norway; ^c^ Department of Psychology, UiT The Arctic University of Norway, Tromsø, Norway

**Keywords:** Acceptance and commitment therapy, fatigue, pain, rehabilitation, group-based

## Abstract

**Purpose:**

To shed a nuanced light on the experiences of taking part in a rehabilitation programme using acceptance and commitment therapy (ACT) for people living with persistent pain and fatigue.

**Materials and methods:**

The ACT intervention, designed by the Pain Clinic of a University Hospital, consisted of six four-hour sessions, each for four groups of 6–8 participants, given over the course of four months. An interdisciplinary research team thematically analysed 13 post-programme individual interviews with people aged 21 to 54 with different symptoms and diagnoses using systematic text condensation.

**Results:**

Participants reported that while illness had led to feelings of loneliness, loss and failure, participating in a safe and supportive group setting led to a nurturing sense of shared community, understanding and learning. Increased self-awareness, self-acceptance and self-compassion were valued outcomes of the programme. Most felt that they had acquired new tools, such as exercises, practices and altered ways of thinking. A few participants were uncomfortable with the sharing practices and felt that the programme brought few benefits for them.

**Conclusions:**

The study indicates the value of a sense of community and experiencing illness as a shared human condition. Learning to see oneself as worthy of self-compassion, suggested in the literature as key to pain rehabilitation, was connected to group validation and ACT-specific sessions. Information, exercises and sharing practices could have been even better targeted and tailored to individual participants.

## Background

Fatigue and pain are common and potentially debilitating symptoms, often seriously impacting people’s lives, regardless of their diagnosis. In our study, we draw on the theoretical foundations of acceptance and commitment therapy (ACT) to approach fatigue and pain as broad and complex phenomena. People suffering from chronic pain and fatigue often also experience emotional distress, depression and anxiety [1]. When biomedical explanations are lacking, patients might feel disregarded and worthless, while health professionals may feel frustrated and helpless [2]. Further, from an existential viewpoint, fatigue and pain are not merely bodily symptoms but demanding life experiences that can unsettle one’s fundamental sense of agency, coherence, and orientation in the world [3]. This understanding is an essential part of the approach to suffering in the ACT model.

Acceptance and commitment therapy (ACT) is a third-wave cognitive behavioural therapy that has shown an effect in the treatment of persistent pain [4,5]. In ACT, the emphasis is on altering the individual’s relationship with pain and distressing thoughts and emotions [4]. By framing these experiences as universal, transient and non-threatening rather than pathological, their avoidance and the struggles involved are thought to decrease. The core mechanism is thought to be enhanced psychological flexibility, through six key processes: acceptance and cognitive defusion, both leading to *openness*, contact with the present moment and developing an ‘observer self’, both leading to *awareness*, and finally, values-based action and commitment, which result in *engagement*. In other words, mindfulness and acceptance processes can lead to psychological flexibility, which can promote behavioural change [6].

In contrast to the large body of quantitative studies about the effects of ACT, there are few qualitative studies. In the late 1990s, the pioneers Steen, Haugli and colleagues [7,8] developed the Vitality Training Programme, an emotion-focused group therapy consisting of ten group sessions over four months, sharing several features and goals with more recent ACT interventions. Zangi et al. [9] collected data from ten focus group discussions with 69 of the 91 patients, two weeks after the intervention. The participants reported that after taking part in the programme, they saw themselves as much more than just patients, acknowledged their emotions such as grief and anger, became more aware of their own needs, felt part of a safe and supportive community and felt acknowledged as credible patients. Several reports from a more recent Norwegian occupational rehabilitation programme based on ACT are interesting and relevant for the present study. In a focus group study with 35 patients interviewed before and after a 3,5 week group-based program, participants described having embarked on a long journey of raised awareness of their goals in life following the programme [10]. All participants in the program were invited to take part in the qualitative study. They appreciated working in a transdiagnostic group, which facilitated focusing on coping with their health problems instead of dwelling on them [11]. However, qualitative studies in this research field are still scarce, and we need more knowledge to understand the breadth and depth of experiences with ACT interventions of patients living with persistent, debilitating symptoms such as pain and fatigue.

This study aimed to shed a nuanced light on the individual experiences of taking part in a rehabilitation programme using ACT for people living with persistent pain and fatigue. We were particularly interested in participants’ experiences with processes of change, and how they felt that the programme facilitated such processes.

## Materials and methods

### Design

This was a cross-sectional interview study with participants who had completed a multimodal transdiagnostic ACT rehabilitation programme for patients with longstanding pain and chronic fatigue.

### Intervention

This study was a part of a national quality improvement initiative funded by the Norwegian health authorities, designed to enhance the co-organization of treatment for patients with long-term pain and chronic fatigue, which often present with overlapping symptoms. As part of this initiative, we developed the current intervention at the University Hospital of Northern Norway.

The ACT intervention consisted of six four-hour sessions, each for four groups of 6–8 participants. It included group conversations and exercises designed to enhance awareness and acceptance of inner experiences, facilitate value clarification, and promote values-based action. The first three sessions took place over the course of three days, the subsequent two sessions one month later over two days, and the final one-day session three months later.

The first (three-day) sessions focused on establishing a safe and supportive group atmosphere while introducing ACT processes through experiential exercises and dialogue. Key topics included cultivating acceptance of unpleasant inner experience, clarifying core life values, and developing individualized, values-based action plans. The second (two-day) sessions emphasised reflecting on participants’ experiences at home with their action plans and deepening their understanding of core ACT processes, such as cognitive defusion and mindfulness skills. The final one-day session was dedicated to consolidating new learning and exploring ways to apply insights gained to daily life. The intervention was conducted by two psychologists experienced in ACT-based group therapy.

### Study population, sampling and recruitment

Participants were recruited from the Pain Clinic and the Physical Medicine and Rehabilitation Centre at the University Hospital of North Norway. The patients had been referred to specialist care by their general practitioners. Patients aged 18–70 years suffering from chronic pain and/or chronic fatigue were invited to participate. Patients were notified about the intervention by clinicians, and those interested received an individual session with further information from one of the instructors. They could then decide to enrol in the group intervention.

### Participants

All 18 participants from three subsequent treatment groups were invited to take part in the interview study. 13 of these said yes. The sample consisted of two men and 11 women, aged 21 to 54 years, with various diagnoses, such as neck pain, myalgia, fibromyalgia, lumbago, tiredness, exhaustion and chronic fatigue syndrome/myalgic encephalomyelitis. Most participants were sick-listed or on work assessment allowance, but some were working part-time or full-time. See the table in the Appendix.

Participants across the three groups attended all scheduled group sessions, except for one participant who missed the final group session but participated in an individual follow-up session. Homework involved engaging in individualized action plans that participants developed during the group sessions. All participants worked on their action plans between sessions.

### Research group

The study was planned and the results analysed by an interdisciplinary research team. Ida Solhaug was a clinical psychologist working at the Pain Clinic at the time and is an associate professor of psychology. She was a co-designer and a co-instructor of the ACT programme and thus had an insider perspective on the intervention. Thor Eirik Eriksen is a philosopher and associate professor specializing in occupational health. He was a teacher of one learning activity during the rehab programme, facilitating a group dialogue targeting acceptance processes. TEE approached the study from a theoretical, phenomenological perspective. May-Lill Johansen (MLJ) is a specialist and associate professor in general practice, skilled in qualitative research methods. She was not involved in the ACT rehabilitation programme. MLJ drew on her experience as a health services researcher and a general practitioner. The different perspectives fostered vivid and fruitful discussions, leading to a variety of interpretations and an emphasis on reflexivity.

### Data collection

To explore the participants’ experiences of participating in the intervention, MLJ conducted semi-structured interviews with them some months after their last session. When contacted by MLJ, they received oral and written information about the purpose of the interview and were asked to sign a consent form. They were invited to choose their preferred interview location, in their home or in MLJ’s office in the university. Interviews concerned illness experiences, daily habits, relations to self and others, work situation and any changes in these domains after the programme. Interviews lasted about one hour, were digitally recorded, and then transcribed and anonymized.

### Analysis

We conducted an inductive thematic analysis within a realist framework to identify patterned meanings in participants’ accounts. All authors first coded transcripts independently, seeking patterns or themes covering various aspects of the participants’ experiences. After agreeing on potential themes, each of us coded further interviews. After coding, we gathered all text relevant to each potential theme. We then made a thematic map of the entire data set and decided which themes to present in this, the first article from the study. For the final revision and refinement, we went back to the coded data extracts and looked closely at the participants’ forms of semantic expression. This led to a reordering and renaming of codes and themes relevant to this report, moving the analysis to a more interpretive level. Thereafter, the first author performed systematic text condensation [12] of the transcribed text pertaining to each theme. This step further clarified which themes were most relevant for this paper.

## Results

Our analysis of the participants’ experiences with the intervention resulted in four themes, which are listed below. To provide a background to these experiences, we first summarise participants’ histories of the period leading up to their illnesses and their treatment journeys.

Most participants described a prolonged period of overachieving and ignoring their own needs, prior to their illness. ‘Being in a rat race’ was a metaphor used. Although many had interpreted their symptoms as warning signals, ignoring them and carrying on had been a common response. Finally, many came to a point where they could no longer persist due to illness. However, many had tried to enter the rat race again after a break.

For most participants, the ACT intervention was just one step in their broader journeys of change. Many had previously engaged in individual therapies, other rehabilitation programmes, or substantial self-help efforts. As such, their experiences reflect the complexity and ongoing nature of personal transformation. From an existential and longitudinal perspective, participating in an ACT intervention should be seen as one of many factors contributing to change, even though some participants did describe the programme in terms of a clear ‘before’ and ‘after’.

### Pushed to explore and share experiences

Participants recalled that early in the programme the instructors asked them serious, challenging questions about their lives: What is important to me? What is important to me when I meet other people? What is important to me when I meet myself? While thinking things through, some found new answers, maybe to their surprise.

Turid: *So you must think properly about: How am I feeling, how do I want to feel, what can I deal with, what can’t I deal with. What’s difficult for me, and what’s quite impossible for me. (…) But if you don’t challenge yourself to ask the difficult questions, nobody will ever answer them (…) You had to think things through again and maybe realize that things have changed since the last time or what I thought things were like, well, they weren’t like that.*

They were then encouraged to share their reflections on the questions with the other participants. Some had never talked to people before about their life situation. At first it felt strange. They could feel vulnerable when talking about themselves in a group setting. It was emotional to share personal stories about the road they had travelled and possible ways out. However, most participants valued the programme leaders pushing them to dig deeper into their personal narratives, values and goals.

Gudrun: *But you’re supposed to talk a bit about yourself, and then you’re in a sensitive situation. And then you might suddenly start crying (…). Because it’s a bit tough, you have to sort of try and tell other people how you’re getting on and how you got to where you are today and what you think the reason is and how you can try and accept it and find tools to work your way out of it or find a balance or move on if you have the chance.*

A few said that being questioned individually about personal issues in a group of strangers felt awkward and compromising. They were confused about the objectives of the programme, as they had expected specific advice on how to live better with their symptoms. However, all participants appreciated the more relaxed talking and laughing with each other in the coffee breaks.

Olivia: *The most useful thing for me was meeting other people in the same situation. I really think the best part of the whole course was the breaks when we sat together and talked about things*.

Bjarnhild: *Because (…) we got to know each other better in the group and I think we benefited more from (…) the breaks* [laughter] *where we could talk about* [personal] *things. Because then the conversation went, was about* [personal] *things, but in a different way, in a more relaxed way (…).*

### The feeling of community alleviated shame

All participants, regardless of their diagnosis, appreciated the group-based nature of the programme, and many considered peer support to be the most valuable part. By openly and trustfully sharing their experiences, the participants realized that they were not alone, and not abnormal, with their symptoms and their suffering. The feeling of being in the same boat gave them relief from worries, self-blame and shame.

André: *So it helped me to join the group treatment and see that if you can’t work, it’s nothing to be ashamed of, and there are lots of people with those kinds of problems. And I used to think I was the only person in the whole world there was something wrong with, you know, for a period.*

Many referred to the programme as a safe place, where participants were seen, understood and looked after, a place where they could be themselves without judgement.

Mona: *It was a nice safe environment. They looked after you (…) you could cry, and nobody said: Oh, stop whining again.*

Being in the group felt good, and although their ailments, ages and life situations were different, they could feel understood by the others in an unusual way. When someone started to say something, the others immediately knew what was coming.

Anne: *It was really so nice (…) if you just mentioned a feeling (…) the others knew what you were talking about (…) we just had to laugh about it many times, because (…) it was like an old marriage where you kind of knew everything about each other, where you could just (…) complete everything they said.*

One person’s sharing of experiences could become new insights for another. In this way, both could feel valued and gain self-esteem. Listening to the others’ stories was also a way of learning about oneself. Participants found that they could have said similar things, but had never thought about the issue in that way.

Turid: *Well, you know, when you hear other people tell their story, you think: But I could actually say the same things* [about myself]. *But I’ve never said them. I’ve never thought about it. It’s never occurred to me that it could be like that or that’s the way it is* [for me too].

### Developing self-compassion and self-care

Recognizing their existential situation as precarious was an important realization by the participants. Many reported having ignored their feelings and bodily sensations for prolonged periods. Living like a robot was one metaphor used.

Turid: *To myself, I hardly existed as a person, or my feelings or … I didn’t have time for them, I hadn’t taken time for them for many years. I’d just sort of pushed them aside, because they were an obstacle, a delay, I just had to … you know.*

Recognition could mean to start feeling the pain and sense the bodily tensions. It could mean opening up to one’s emotions and grieving for lost abilities and opportunities.

Gurdrun: *(…) a sad feeling. Because you’re not the same as before. Yes, you know, feeling sad about losing all that and not being able to take extra shifts and just go out with my colleagues straight after work on a Friday and … I can’t do that any more.*

Most participants felt that the programme had led to changes in their lives. An improved relationship to oneself was pointed out as particularly important. As ill people with a malfunctioning body, many had felt like failures. It felt good to get to know oneself better, see oneself with new eyes and start to like and care for oneself again, and it led to more compassion for others.

Sophie: *So slowly but surely, I feel that I’ve managed to change my focus and my view of myself. Because you feel like such an awful failure as a human being when you get ill and your body doesn’t work (…) Starting to like myself again. I reckon that must be the most important thing.*

Caring for oneself could mean learning what is good for you, and then choosing wisely: What do I like, what do I want and what makes me happy? The participants mentioned pursuing and enjoying bright moments and being present here and now. It felt good to wind down their expectations of keeping perfect order and control at home. Even doing nothing could sometimes be fine.

Veronica: *So maybe I’ve learned it a bit better in practice now, finally how to take care of myself (…) Now I hardly ever push the cat off my lap when she jumps up. Then I just sit there and we have a nice cuddly time together. And then other things have to wait. I think the idea is that I should just sit there and enjoy life.*

### New tools, new thoughts, new twists

Many participants mentioned that the programme gave them tools to help them handle their symptoms better. Examples were mindfulness exercises and taking time-outs. Several found that the programme made them more conscious about sensing the present moment, such as when out for a walk. Others felt that most tools were not suitable or useful for them.

Sverre: *We got some tips, some hints, some exercises (…) when I went home, I had a few more tools in my toolbox to manage my life.*

Mona: *I thought the breathing (…) that’s when I could relax (…) I wish I could have taken their voices with me too, when they said do this and do that. Because they had such a calm voice.*

Emilie: *I haven’t taken any – as far as I can remember – taken any home with me. I didn’t feel that any of the exercises were right for me.*

Managing everyday life could feel easier after having learned to rethink one’s capacities: if one’s current capacity was 50% of one’s previous capacity, then 50% would now be the new 100%. Many participants found this realization helpful to avoid relapses of guilt and self-degradation. Understanding how they could lower their demands on themselves was perceived as important.

Inger: *I sort of try like this: OK, I might be able to do a bit of something (…) and I buck myself up (…). If I can’t do all of it, at least I’ve tried. So that’s exactly what I felt did me good. (…) I don’t think I would have thought like that if I hadn’t been on that course.*

Grete: *ACT (…) made me realize that I might never be the same again. But you sort of have to find a new path. Maybe it’s good to have 20% on your battery. I mean, I’m sure you can function well on that. You just have to find that path, that’s what I think.*

Participants talked about learning to see things from another point of view. Sorting things out in their heads, finding pegs to hang things on, and learning new twists on old thoughts were seen as helpful. Just swapping one word for another could help. Instead of saying I must, or I should, they were learning to say I want to, or I wish, and to prioritize. Thinking anew could also mean changing their goals. They had also learned to meta-think and recognize thoughts and worries as just that, not reality. Several referred to insights like ‘a thought is just a thought’ and ‘I am much more than my illness’ as new ways of meta-thinking.

## Discussion

All the participants appreciated the group-based character of the programme. There seemed to be instant recognition in the group when one participant shared stories of living with persistent symptoms, which resulted in mutual validation. This sense of recognition was evident across participants, regardless of diagnosis, highlighting a common phenomenological experience of living with a body affected by pain and fatigue. Recognition and validation of their suffering and struggling by other participants meant that isolation and loneliness were replaced by a feeling of community and self-validation, as also highlighted by others [13–16]. Social learning took place through sharing illness stories and interpretations of them. These group processes seemed to nurture hope and planning for life changes. Steen and Haugli [7] also found that people who disclosed personal feelings acknowledged their own situation and enhanced their self-awareness, while members of the group listening to them could recognize themselves in the stories told. The significance of the feeling of group community was also emphasized by Zangi et al. [9], as ‘a basis for exploring and expressing how they really felt in a way that they had not experienced before’ [p. 422].

Working in groups is not ACT-specific, but a generic component of many rehabilitation programmes. According to a theoretical model by Day et al. [17], the environment of an intervention, e.g. improved social support, a strong therapeutic alliance and strong group cohesion, is thought to influence all other modes of the programme. The authors state: ‘Research suggests that group factors, such as cohesion or social learning, (…) can become agents of change in and of themselves’, and that such group factors might form the basis of all successful psychosocial interventions [17, p. 695]. The mixture of ACT-specific and generic elements was regarded as favourable in several studies, and particularly highlighted in Thompson et al. [18].

While participants had neglected themselves to a considerable extent, validation by other participants and instructors could help them to see themselves as worthy of self-compassion. This insight was often accompanied by strong emotions, such as tears of sadness and tears of relief. This could have been a reaction to the gap between their current life situation and where they would actually like to be [19]. Notably, Bremander et al. [13] and Aymerich et al. [14] suggested that the development of self-compassion might be a key process within pain rehabilitation.

Grieving for lost capabilities and opportunities could be a step towards accepting feelings of profound losses and reorienting oneself towards new goals [9]. Gustafsson et al. [20] described how validation from the group made participants’ emotions of shame change to self-respect. Resisting these important sharing practices, as did some of our participants, might have protected them against painful experiences, but also against validation and motivation for change. Other studies, e.g. Nøst et al. [15], have also shown that some participants found it challenging to share their stories with others. Indeed, we all have a certain resistance to discovering and changing longstanding unhealthy patterns in our lives [8].

In the interviews, we did not systematically ask about experiences of the six core processes of ACT. Although our coding was not theory-driven, several themes align conceptually with ACT processes, including acceptance (reframing ‘the new 100%’), values and committed action (reprioritising daily choices), and present-moment awareness (mindfulness practices), which are consistent with the psychological flexibility model. On the way to acceptance, many found it helpful to see their current capacity as their new 100%. Participants also considered it a sign of acceptance that they had begun to sense how they felt at that moment and then planned their day accordingly.

The programme contained mindfulness exercises to facilitate contact with the present moment. As in previous studies, e.g. Gismervik et al. [19], some understood these as relaxation practices, while others connected them to being more aware, seeing presence also as an act of self-care. Increased awareness of relations to self and others was a main theme in the programme, which resonated well with most participants. Exercises helped to encourage awareness of self and taking a meta-perspective on self. However, some of the participants did not find the exercises helpful.

Clarifying and connecting to their values, and then using this to commit to behavioural changes, was an aim of the programme that most participants appreciated. However, as the programme was just a relatively short stop on their journeys, they knew that the road ahead would be long and winding. Steen and Haugli [8] note that becoming aware of unhealthy patterns in our lives takes time, while reinterpreting and eventually changing them takes both time and practice.

### Strengths and limitations

Unlike most studies on group-based rehabilitation programmes, we chose individual interviews instead of focus group discussions with participants. In individual interviews, the conversation between researcher and participant is more personal and goes into greater depth and detail to explore a topic [21]. Individual conversations are more likely to capture and explore diversity, including negative experiences and critical opinions. We found that these strengths of individual interviews were suitable for our research aim.

The interviewees varied in terms of age, gender, illness duration, symptoms, diagnosis and previous treatment. As the interviews took place several months after the last treatment session, the participants could assess the intervention in their life context. However, interviewing them both before and after the treatment period could have further illuminated what each of them learned from this specific programme. The interviews could also have included more questions about experiences with the ACT-specific content of the programme.

Based on our discussion with the international literature, we think our results regarding the perceived benefits of ACT-facilitated group processes are transferable to other group-based rehabilitation programmes with ACT, particularly for patients with persisting physical symptoms.

## Conclusions

This study adds to the qualitative literature on how people suffering from persistent pain and fatigue have experienced a multimodal rehabilitation programme using ACT. For individuals with self-blame and shame, being invited to join a supportive peer group seemed to have a healing effect. Being guided by ACT on the path from self-neglect to self-compassion was regarded as vital for their onward journeys. As in other studies, not all participants were comfortable with the sharing practices, and not all found the programme beneficial to them.

The generic group effects, strikingly similar to other studies, indicate the value of a sense of community and experiencing illness as a shared human condition. The process of seeing oneself as worthy of self-compassion, suggested in the literature as key to pain rehabilitation, was connected both to group validation and to ACT-specific sessions. Information about the programme, exercises and sharing practices could have been even better targeted and tailored to individual participants.

## Supplementary Material

Table Participants ACT interview study.docx

ACT intervention programme.docx

Semistructured interview guide.docx

## Data Availability

The data that support the findings of this study are available upon reasonable request from the first author. The data are not publicly available due to privacy or ethical restrictions.
