# Supplementary material for: Acceptance and commitment therapy in rehabilitation for chronic pain and fatigue: a qualitative interview study with patients
Source: Scand J Prim Health Care. 2026 Jan 6;44(1):2608121. doi: 10.1080/02813432.2025.2608121 (PMC12781931; doi:10.1080/02813432.2025.2608121)
Supplement: Table Participants ACT interview study.docx [file IPRI_A_2608121_SM9959.docx]

Participants ACT interview study

| Participant  number | Age group | Diagnoses | Place of residence |
| --- | --- | --- | --- |
| 1 | 50 - 60 | Myalgia | Rural |
| 2 | 20 - 30 | Myalgia | Town |
| 3 | 50 - 60 | CFS/ME | Rural |
| 4 | 50 - 60 | CFS/ME | Rural |
| 5 | 50 - 60 | Myalgia | Town |
| 6 | 40 - 50 | Fibromyalgia | Town |
| 7 | 20 - 30 | Tiredness | Town |
| 8 | 20 - 30 | Fibromyalgia | Town |
| 9 | 30 - 40 | Tiredness | Town |
| 10 | 40 - 50 | Exhaustion | Rural |
| 11 | 40 - 50 | Exhaustion | Rural |
| 12 | 40 - 50 | Fibromyalgia | Town |
| 13 | 40 - 50 | Fibromyalgia | Town |
