# Supplementary material for: Acceptance and commitment therapy in rehabilitation for chronic pain and fatigue: a qualitative interview study with patients
Source: Scand J Prim Health Care. 2026 Jan 6;44(1):2608121. doi: 10.1080/02813432.2025.2608121 (PMC12781931; doi:10.1080/02813432.2025.2608121)
Supplement: ACT intervention programme.docx [file IPRI_A_2608121_SM9958.docx]

| **ACT group intervention for patients with**  **long-term pain and fatigue** | **First 3-day sessions^[[1]](#footnote-1)^** | | | **2-day sessions**  **one month later** | | **1-day**  **session**  **three months later** |
| --- | --- | --- | --- | --- | --- | --- |
|  | **Day 1** | **Day 2** | **Day3** | **Day 4** | **Day 5** | **Day 6** |
| 45 min session | Introduction  Establishing group | Mindfulness training and dialogue | Mindful movement | Mindfulness training and dialogue | Mindful movement | Mindfulness training and dialogue |
| 45 min session | Biopsychosocial lecture on pain and fatigue with doctor^[[2]](#footnote-2)^ in pain rehabilitation | Defusion exercises and dialogue | Values clarification  work | Sharing experiences with action plan at home | Accepting pain.  Defusion.  “My new 100 %”. | Sharing experiences from home period |
| 45 min session | Mindfulness and mindful movement.  From autopilot to conscious choice. | Values clarification  work. | Commited action | Former patient and  yogateacher shares experiences with fatigue and mindfulness | Values clarification  work | Values clarification  work.  Committed action. |
| 45 min session | Introducing ACT principles. “Creative hopelessness”. | Group dialogue with philosopher TEE on concept and practice of acceptance | Expressing action plan through “Bulls Eyes”-exercise on the floor | Acceptance work | Making and sharing new action plan | The way ahead.  Evaluating the group participation |

1. The intervention was conducted in a region of Norway characterized by large geographical distances. Due to the long travel times for participants, we opted for an alternative structure to the traditional weekly sessions. Instead, sessions were clustered over several consecutive days, with months-long intervals between these clusters. [↑](#footnote-ref-1)
2. The intervention was facilitated by two psychologists with extensive experience in delivering group-based ACT interventions, one of whom was the third author (IS). Additionally, a medical doctor, a philosopher (second author TEE), and a former patient were invited to contribute to specific sessions as appropriate. All contributions were carefully designed to align with ACT principles. [↑](#footnote-ref-2)
