# Supplementary material for: Acceptance and commitment therapy in rehabilitation for chronic pain and fatigue: a qualitative interview study with patients
Source: Scand J Prim Health Care. 2026 Jan 6;44(1):2608121. doi: 10.1080/02813432.2025.2608121 (PMC12781931; doi:10.1080/02813432.2025.2608121)
Supplement: Semistructured interview guide.docx [file IPRI_A_2608121_SM9957.docx]

**Interview guide**

The interview guide is semi-structured, dealing with some overarching topics. We strive for openness and sensitivity regarding new topics arising from the conversation. The questions below are tentative. They will be formulated in simple, everyday language. We are inviting “thick”, experience-based stories and descriptions.

**Health:**

If you were to provide me with an overview of the history of your illness from the beginning until today, what would I need to know?

How do you experience your illness today?

How do you understand your diagnosis, and how do you relate to it?

What does it mean to be in “good health”, in your opinion?

Has your experience of your illness changed since you participated in the rehabilitation programme? In which way?

What do you think contributed to a change or a non-change?

Have you experienced that your habits or ways of acting have changed?

How do you manage stress in your life?

**Relationship to self**:

How would you describe yourself in a few words?

How do other people often describe you?

Has your illness contributed to changes in your understanding and experience of yourself? How?

Has participating in the programme influenced or changed how you experience and see yourself? How?

**Relationships:**

Has your illness and your way of managing it influenced your relationship with your family? How?

Has participating in the programme influenced or changed your way of relating to other people? How?

**Working life/student life:**

Has your illness influenced your participation in working life/student life? How?

Has taking part in the programme influenced or changed your relationship with working life/student life?

**Experiences of the rehabilitation programme:**

How did you experience taking part in the programme?

What was the most essential point you learned or took home with you?

Did anything surprise you during the treatment process?

Was there some part of the programme that was not helpful to you?

What could have made the treatment more helpful to you?

**Change over time:**

The rehabilitation programme lasted half a year. Did you experience any changes underway between the seminars? If yes, how would you describe this process of change?

Now X months have passed since you finished the programme. Do you feel that you have taken something from the programme that has influenced the way you live your life today? Please elaborate. If you have not, what could be the reason for that?
